# Planet earth calling: unveiling the brain’s response to awe and driving eco-friendly consumption

**DOI:** 10.3389/fnins.2023.1251685

**Published:** 2023-10-02

**Authors:** Meiling Yin, Eun-Ju Lee

**Affiliations:** ^1^Business School, Sungkyunkwan University, Seoul, Republic of Korea; ^2^Neuro Intelligence Center, Sungkyunkwan University, Seoul, Republic of Korea

**Keywords:** eco-friendly, climate, awe, fMRI, self-awareness processing, external attention processing

## Abstract

Eco-friendly consumption is important for solving climate crisis and moving humanity toward a better future. However, few consumers are willing to pay premiums for eco-friendly products. We investigated the psychological and neural factors that can increase eco-friendly consumption. We propose an experience of awe, in which the individual self is temporarily attenuated as the importance of beings other than oneself increases. Behavioral (Study 1) and functional magnetic resonance imaging (fMRI; Study 2) experiments were conducted to explore the awe mechanisms through which climate crisis messages lead to eco-friendly consumption. In Study 1, we found participants felt awe when exposed to climate crisis messages, and their choice of eco-friendly consumption increased. In Study 2, we found that when individuals were exposed to messages depicting the climate crisis (as opposed to a control stimulus), their brains exhibited a lower level of activation in the self-awareness processing and a higher level of activation in external attention processing areas. These results suggest that the awe experience plays an important role in promoting eco-friendly consumption. Marketing must evolve from satisfying basic individual needs to a high level for the well-being of humanity, the planet, and the biosphere. This study sheds light on our understanding of human perceptions of the climate crisis and suggests an effective communication strategy to increase individuals’ eco-friendly actions.

## 1. Introduction

As environmental problems such as resource scarcity, depletion of the ozone layer, climate change, and species extinction have emerged, sustainable consumption behaviour has become increasingly important. However, despite researchers’ warnings about climate change, institutions that dominate the planet continue to extract resources from nature (Hassan et al., 2019). Sustainable behaviour requires individuals to accept the need for sacrifice to support the greater good (Bangsa and Schlegelmilch, 2020; Boenke et al., 2022). Although not all individuals contribute equally to climate protection, all individuals share the benefits of protecting the environment (Hasson et al., 2010). Eco-friendly behaviour provides tremendous benefits to both human and the planet’s well-being, but maximizing individual interests often makes more sense to the individual in the short run. Therefore, to promote sustainable consumption, humans must form cooperative, mutually adaptive, and reciprocal relationships with nature.

Visual representations of climate change can help the public understand it by connecting abstract concepts related to climate change with natural experiences (Klöckner, 2015). Many firms use green logos and natural images to promote sustainable consumption. However, it is unclear whether these visual representations affect consumers’ sustainable behaviors and, if so, what the underlying mechanisms are. Some researchers say that social desirability or selfish motivation induces eco-friendly purchasing (Trudel et al., 2020; Yue et al., 2020), whereas others state that it is driven by the environment itself (Cavanaugh et al., 2015; Yan et al., 2021). Therefore, we consider the following question: when climate change is visually expressed, which of the two, self-enhancement or the small self, acts as a mechanism to increase eco-friendly consumption? It is important to examine the relationship between self-perception and eco-friendly consumption because self-theory is frequently mentioned in luxury consumption but limited in eco-friendly consumption.

A difference exists between self-enhancement and the small self (Keltner and Haidt, 2003). Self-enhancement is a feeling of personal accomplishment at the individual level (Pfeffer and Fong, 2005) whereas a small self is an experience of awe in which the individual self is temporarily weakened (Campos et al., 2013). These differences drive consumers to make different choices in their sustainable choices. Given this, we propose that awe promotes sustainable behaviour. Self-enhancement is the key driver of consumers’ proclivity toward luxury consumption (Stathopoulou and Balabanis, 2019). However, we believe that adopting a self-transcendent perspective, such as awe, can promote sustainable behaviour because prosocial behaviour benefits other individuals.

Previous studies have often used self-report questionnaires to explain consumer perceptions and behaviors. However, people often earn high scores for their environmental values but face a “value-action gap” in terms of their purchases (Gupta et al., 2022). Therefore, the psychological and neural mechanism of “awe,” being conscious of Earth and Mother Nature instead of self-centered consciousness of the surroundings and the world, is investigated in this article using functional magnetic resonance imaging (fMRI). Specifically, the purpose of this study was to elucidate the neural and psychological mechanisms that are predicted to increase the sense of awe toward Earth and Mother Nature, moving individuals away from the egocentric state discussed earlier. The response to visual stimuli related to the Earth’s environment and climate crisis was explored using fMRI. Additionally, the purpose was to behaviourally investigate how this increased sense of awe and reduced sense of self, as described above, are significantly related to eco-friendly consumption.

To clarify this phenomenon, we focused on processing climate-change messages and eco-friendly consumption. First, we examined the psychological processes that occur when individuals viewed messages related to climate change. We hypothesized that participants would experience awe accompanied by reduced activation of the self-awareness path and increased activation of the external attention path when viewing climate change messages. Second, we examined the relationship between messages about climate change and eco-friendly consumption, proposing that a sense of awe increases consumers’ environmental awareness and eco-friendly consumption behaviour. Finally, we examined the relationship between neurobiological mechanisms and future behaviour. These findings will help to understand in depth the intensity of brain activation and polarized consumption and reveal individual psychological mechanisms related to climate change.

## 2. Literature review

Pro-environmental behaviour refers to consumer actions that lower adverse environmental impacts across the lifecycle of a product, behaviour, or service (White et al., 2019). Previous studies have suggested different antecedent variables that affect prosocial behaviour. One stream of research suggests that individual motives, such as egocentric or status-seeking motives, promote sustainable consumption (Gupta and Ogden, 2009; Griskevicius et al., 2010). Other research streams have promoted sustainable consumption, focusing on social norms, such as moral foundation, social value orientation, and environmental responsibility (Wu and Yang, 2018; Culiberg et al., 2022). In the context of consumption, consumers make decisions based not only on self-motivation or values but also on social interactions and the influence of others (Aagerup and Nilsson, 2016). Therefore, choosing eco-friendly products is necessary for consumers to inform others about their identities (Gao et al., 2021). Individuals often choose eco-friendly consumption to gain social recognition because it reminds them of their social responsibility and creates a positive image for their peers.

More recently, emotions toward the “environment” itself have become tools to promote sustainable consumption. Emotions are largely divided into positive and negative emotions (Liljander and Strandvik, 1997). Negative emotions include guilt and shame, which affect sustainable behaviour (Rees et al., 2015). However, in terms of positive emotions, research has focused on interest in, connections to, and empathy for nature, which promote sustainable consumption (Milfont and Duckitt, 2010; Lee et al., 2020; Casalegno et al., 2022). This is a process in which interest in the individual decreases and interest in others and nature increases. Previous marketing research has focused on self-interested emotions, this notion of the non-self seems contrary to modern individuals’ main motivations and desires for the self. However, reducing the self to a minimum is important for overcoming the most problematic or undesirable aspects of self-awareness, because it means prioritizing the well-being of the natural world and the surrounding environment, including other life forms, rather than focusing solely on improving oneself (Baumeister and Boden, 1994). Reducing the self can be viewed as a departure from self-awareness and a more promising long-term goal (Frantz et al., 2005).

### 2.1. Climate change and awe

Modern society attaches importance to personal values (e.g., achievement and power), resulting in an expansion of the self (Piff et al., 2015). However, the expansion of the self reduces self-sacrificing behaviour (McConnell and Jacobs, 2020). To enact significant changes in decision-making processes and behaviors, it is necessary to change self-awareness (Baumeister and Boden, 1994).

Awe is an other-oriented emotion and a shift of attention from a self-centered perspective to that of larger beings such as nature and the Earth (Perlin and Li, 2020). We propose that exposure to larger entities can induce feelings of awe. When exposed to environmental cues, such as landscapes, humans form a strong sense of connection to the environment and diminish their focus on the self (van Elk et al., 2019). Several studies have reported that exposure to the natural environment leads to a perception of vastness and dramatically expands an individual’s usual frame of reference (Shiota et al., 2007; Campos et al., 2013). This vastness is associated with the sense that one is part of something larger than oneself (Piff et al., 2015). When placed in front of a larger object, people expand their self-definitions by describing themselves, not as “special” or “one of a kind,” but as “a person” or “an inhabitant of Earth,” challenging their own frames of reference to include something larger than themselves (Shiota et al., 2007). When exposed to nature, people alter their self-concepts and experience awe (Shiota et al., 2007). Therefore, we propose the following hypothesis:

H1: When exposed to climate crisis messages, individuals experience a sense of awe.

### 2.2. Neural representation of the climate change effect

**Figure 1:**
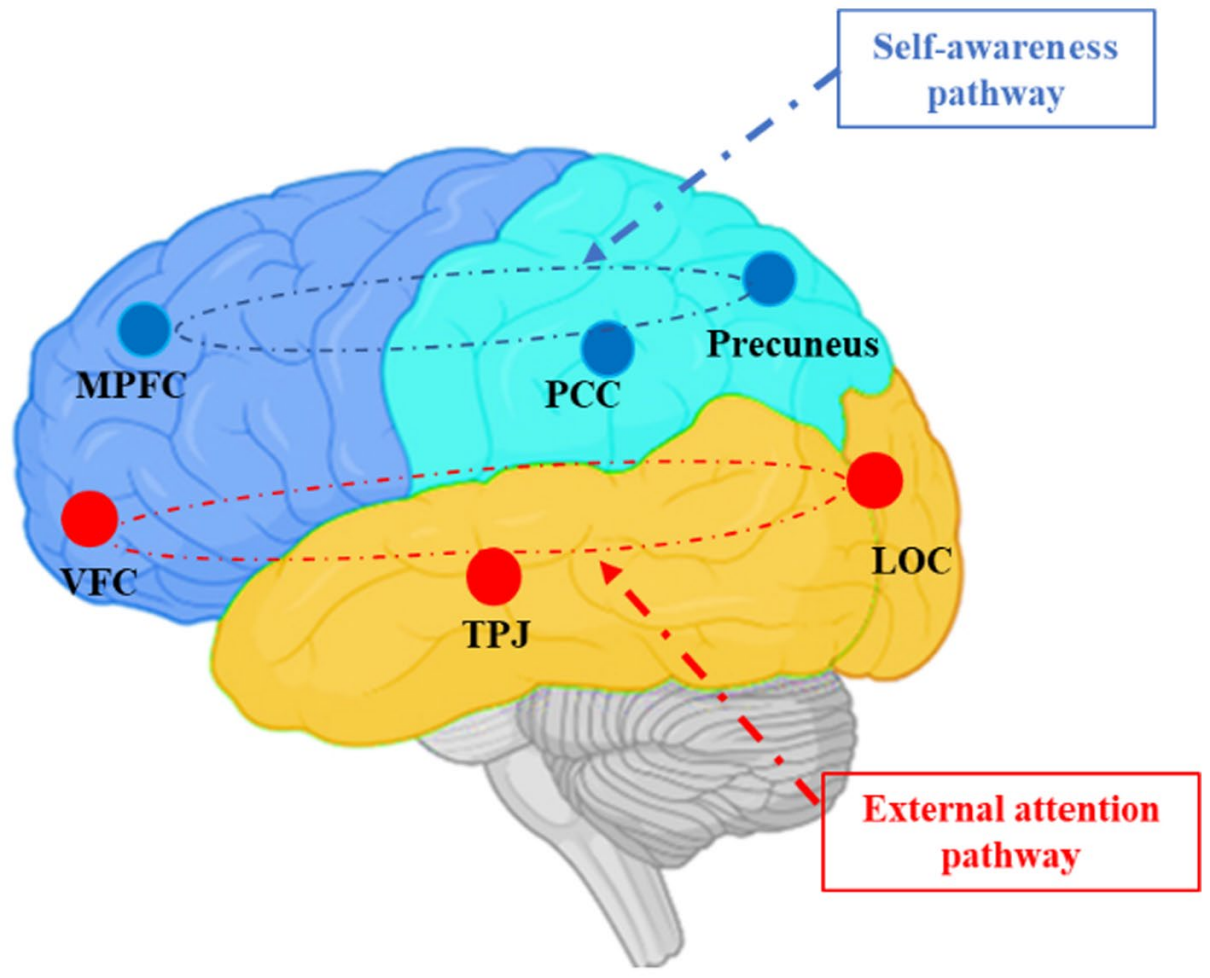
The blue paths indicate the self-awareness process, including the medial prefrontal cortex (MPFC), precuneus, and posterior cingulate cortex (PCC). Red paths indicate the stimulus-driven external attention process, including the ventral frontal cortex (VFC), temporoparietal junction (TPJ), and lateral occipital cortex (LOC).

Neuromarketing can uncover processes hidden in a consumer’s “black box” (Yun et al., 2021). Individuals’ perceptions of the environment can be identified through brain activation, while individuals view images of climate change. The human brain is closely related to self-referential activity at rest (Utevsky et al., 2014), but stimulation by the external environment leads individuals to understand the environment and engage in goal-directed behaviors (Marsh and Legerstee, 2017). There are two distinct anticorrelated paths: a “self-awareness” path and an “external attention” path (Vanhaudenhuyse et al., 2011). The self-awareness path encompasses the medial prefrontal cortex (MPFC), precuneus, and posterior cingulate cortex (PCC), and is engaged in self-referential processing (Demertzi et al., 2013; Davey et al., 2016). The external attention path encompasses the ventral frontal cortex (VFC), temporoparietal junction (TPJ), and lateral occipital cortex (LOC), and is engaged in the process of external detection (Corbetta et al., 2008). We aimed to identify the two neural pathways in the consumer brain that lead to eco-friendly consumption: reduced self-awareness and increased external attention (Figure 1).

#### 2.2.1. The self-awareness processing network

The core components of the default mode network (DMN) are the MPFC, precuneus, and PCC (Utevsky et al., 2014). The DMN is activated when one is engaged in spontaneous thinking (e.g., self-referential processing and mind-wandering) or at rest and deactivated when one is participating in cognitive tasks (Parmar et al., 2022). Specifically, the MPFC has been associated with the processing of self-related information (Kircher et al., 2002; Davey et al., 2016) while the PCC is primarily involved in getting caught up with one’s experiences (Brewer et al., 2013).

Existing studies indirectly support the idea that awe reduces the activation of self-referential areas in terms of egocentric awareness, rest, and spatial processing. The functional core of the DMN responds to a wide range of cognitive processes and reduces self-salience (Zhang and Chiang-shan, 2012). Garrison et al. (2014) found that DMN deactivation is associated with loving kindness and a lack of present-centered awareness. Lou et al. (2004) found that activation of the anterior precuneus and PCC decreased as self-referencing decreased when referring to the self, one’s best friend, and a neutral person. Vogeley et al. (2001) found bilateral precuneus activation when participants read short stories written in the first person which was reduced when they read stories written in the third person. Lebedev et al. (2015) reported that ego dissolution was accompanied by reduced DMN activation. Previous studies on spatiotemporal processing reported that allocentric spatial coding deactivates the DMN (Saj et al., 2014; Ganesh et al., 2015). Therefore, we propose the following hypothesis:

H2: When exposed to climate crisis messages, the MPFC, precuneus, and PCC exhibit decreased activation responses.

#### 2.2.2. The external attention processing network

The human brain visually captures and recognizes the surrounding environment within milliseconds (Liu et al., 2009). The external attentional network is central to the ability to recognize and interact effectively in a dynamic environment (Schultz et al., 2008). The attention network enhances the responses during target detection. Regions of the stimulus-driven external attention network include the TPJ, LOC, and VFC. When attention shifts to a new source of information, an environment-focused network is activated.

The occipitotemporal region, the functional core of external attention processing networks, encodes information about upcoming and predicted movements and engages in goal-directed behaviors (Caspers et al., 2010). Recent social cognitive research has shown a relationship between the meaning of behavioral stimuli and LOTC activity. Vry et al. (2015) reported that occipitotemporal activation occurs in imitation tasks in which participants monitor the behaviour they see, which they then match their own motor activity. Martin et al. (1995) demonstrated LOTC activity in a task that required participants to generate an appropriate verb matching an object represented by a name or picture. Shortly, activity in the LOTC areas involves preparing for and executing explicit, goal-directed movements (Lingnau and Downing, 2015), suggesting that the occipital lobe is useful in understanding our surroundings and acting cooperatively. Therefore, we propose the following hypothesis:

H3: When exposed to climate crisis messages, the occipitotemporal areas show increased activation response.

### 2.3. Eco-friendly consumption

Eco-friendly behaviour is conscious actions performed by an individual to lessen the negative effects of human activities on the environment and/or enhance the quality of the environment (Jensen, 2002). While many consumers agree on the causes of environmental degradation, when a practical decision must be made between using environmentally friendly or less expensive products, the choices often do not lead to sustainability (Joung et al., 2014). To encourage prosocial behaviour, it is necessary to understand its causes.

Understanding the self is central to the goal of promoting eco-friendly consumption, as diminished self-importance and increased interest in others and nature are positively associated with a prosocial disposition (Caprara et al., 2012; Boer and Fischer, 2013). Several studies have investigated the relationship between diminishing attention to the individual self and environmentally friendly behaviors. Van Elk et al. (2019) reported that a feeling of awe reinforces prosocial tendencies, and Evans et al. (2013) found that individuals who translate their attention to others engage in sharing environmentally friendly behaviors. Additionally, McCullough et al. (2002) reported that people who feel part of a larger being, such as humanity and nature, also tend to report increased prosociality because of their diminished emphasis on the self and their own interests, and a shift to attending to the larger entities which they are part of.

However, self-enhancement values, including increased evaluations of power and achievement, are negatively correlated with prosocial tendencies (Caprara et al., 2012; Boer and Fischer, 2013). Self-centered people focus on values that maintain or improve the quality of their own lives, advocate environmental exploitation, and are generally not willing to make sacrifices to purchase eco-friendly products (Fortuna et al., 2021).

Environmental exposure is characterized by reduced engagement in self-referential processing and positively correlated with eco-friendly consumption (Collado et al., 2015; Whitburn et al., 2019; Martin et al., 2020). Several studies have reported that perceptually vast stimuli reduce focus on the self and its concerns (Bockelman et al., 2013; Campos et al., 2013). When immersed in environmental stimuli, individuals increase not only their moral considerations (Weinstein et al., 2009) but also their tendency to be generous and helpful (Zhang et al., 2014). The psychological mechanism underlying the effect of climate change on eco-friendly consumption causes individuals to move beyond self-interest by shifting their focus from themselves to others and the outside world (Piff et al., 2015). Taken together, these findings indicate that, to increase eco-friendly behaviour, one must place less importance on oneself and one’s own interests, and more on something that is larger than oneself. Therefore, we propose the following hypothesis:

H4: Individuals’ eco-friendly consumption increases when exposed to climate crisis messages.

H5: Awe mediates the effects of climate crisis messages on eco-friendly consumption.

## 3. Materials and methods

### 3.1. Study 1

#### 3.1.1. Participants

This study explored the psychological mechanisms of climate change messages and their effects on eco-friendly consumption. The survey was conducted using Prolific, and participants were recruited globally. The first page of our survey described the purpose of the study and ensured the anonymity of the responses provided during the experiment. Respondents who agreed to participate in the experiment were randomly assigned by the Prolific software to one of two conditions—climate or control—and were shown messages related to their respective conditions. The one hundred and twenty-three participants had a mean age of 32.8 years (SD = 16.4 years). Sixty-one participants (35 women and 26 men) were in the climate condition, and sixty-two participants (36 women and 26 men) were in the control condition.

#### 3.1.2. Stimuli and procedure

**Figure 2:**
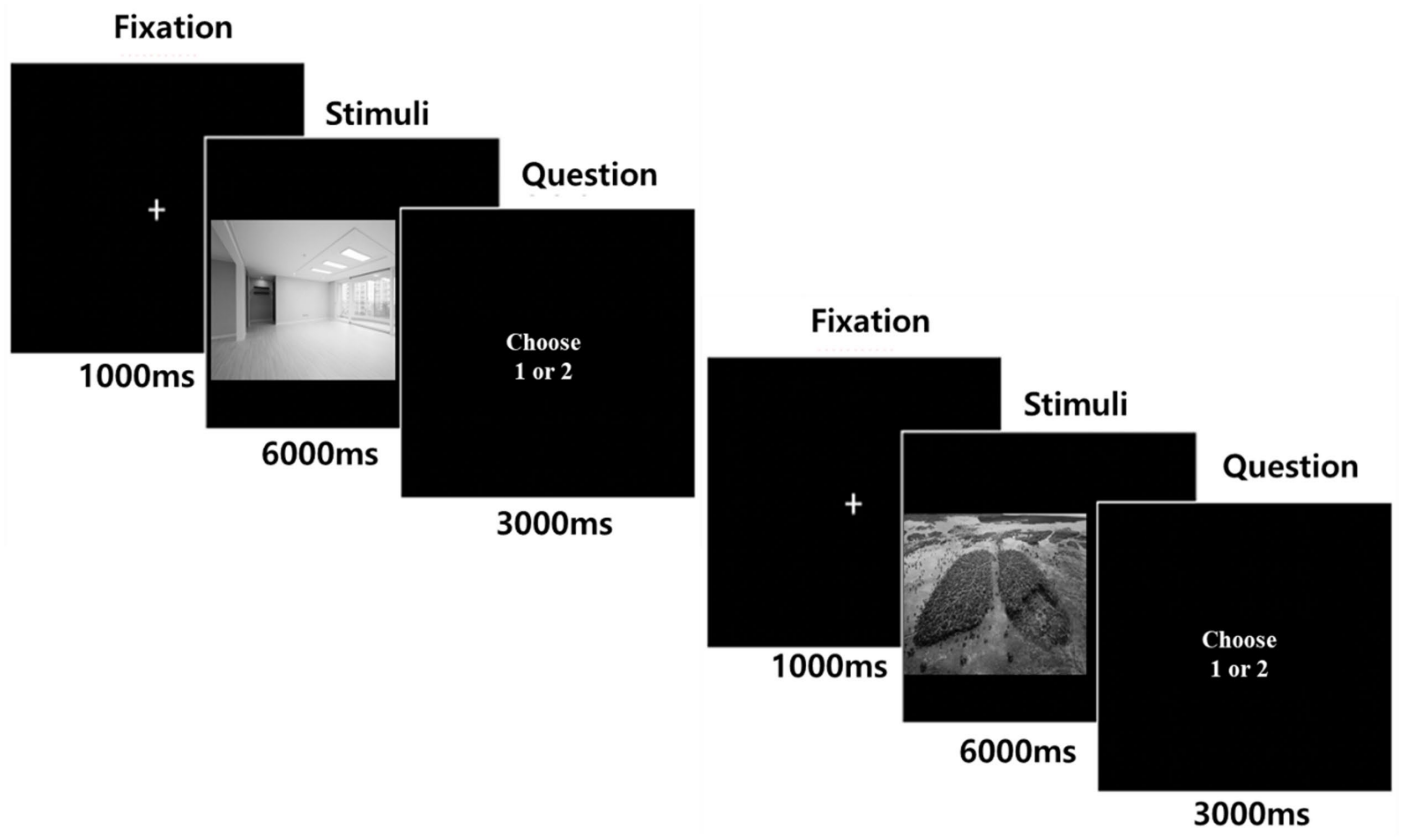
Experimental paradigm.

The behavioral experiment had a single-factor (climate vs. control) between-subjects design (Figure 2). At the beginning of the experiment, the participants read a brief introduction to the experiment and viewed images related to that condition. The images in the control condition were living rooms, whereas the images in the climate change condition were related to the climate crisis, such as climate-related disasters and extremes, ice and related icons, and drought and denuded landscapes. These images reflected climate change, similar to the photos used by Leviston et al. (2014). There were 10 images per condition.

Following each image, we measured the participants’ arousal and the degree to which it was related to climate change. We measured awe in terms of two facets: a sense of the vastness of the self and a sense of self-diminishment (Piff et al., 2015, see Appendix). These 10 items formed a highly reliable index of awe (Cronbach’s alpha = 0.89). At the end of each condition, participants were asked to choose between 10 pairs of products—one eco-friendly and one conventional–at various prices (low to high) in different categories (Lee et al., 2014; Yin et al., 2022). The eco-friendly consumption score is defined as the ratio of respondents who chose eco-friendly products. After the behavioral experiment, the participants were paid $1 for their participation.

#### 3.1.3. Results

After being assigned to each condition, participants underwent a manipulation check for each condition. Participants reported that climate images were more related to climate than control images (M_control_ = 3.73 [SD = 2.12], M_climate_ = 4.82 [SD = 1.86], *t* [1, 121] = 3.04, *p* < 0.01) and that images from the two conditions did not differ in arousal levels (*p* > 0.05). However, the level of awe that the participants felt differed significantly between the climate and control conditions. The sense of awe the participants felt was increased in the climate condition compared to the control condition (M_control_ = 3.29 [SD = 1.31], M_climate_ = 4.67 [SD = 1.15], *t* [1, 121] = 6.16, *p* < 0.001). Therefore, H1 is supported.

The *t*-test analysis with eco-friendly consumption as a dependent variable showed that eco-friendly consumption selection was significantly higher under climatic conditions (M_control_ = 0.58 [SD = 0.27], M_climate_ = 0.72 [SD = 0.20], *t* [1, 121] =3.22, *p* < 0.01). Thus, the climate change message increased eco-friendly consumption, supporting H4.

We also tested the mediation model using the bootstrapping procedure outlined by Preacher and Hayes (2008). Significant mediation through awe existed if the 95% confidence interval did not include zero. The direct effect of climate conditions on eco-friendly consumption was not significant when the mediator was included in the model (Effect = −0.08, CI [−0.17, 0.02]). The indirect effect of climate conditions on eco-friendly consumption was significant when the mediator was included in the model (Effect = −0.24, CI [−0.47, −0.04]). These results indicate the effects of climate messages on eco-friendly consumption through awe. Therefore, H5 is supported.

#### 3.1.4. Discussion

In the behavioral study, participants felt a greater sense of awe in the climate conditions and showed increased eco-friendly consumption. Our results are consistent with those of previous studies, showing that exposure to climate change enables people to behave sustainably (Lee et al., 2020; Yin et al., 2022). This indicates that our experimental design could induce a feeling of awe. Future research is required to better understand the psychological processes of individuals during their exposure to climate change. To investigate the physiological mechanisms during exposure to climate change, we used fMRI to examine the participants’ hidden psychological processes.

### 3.2. Study 2

#### 3.2.1. Participants

We conducted fMRI experiments using climate images as stimuli to look for neural evidence in clues to climate change. All contents and procedures of the experiment were conducted with the approval of the Institutional Review Board (IRB; IRB no. 2021–12-018), and all participants provided written informed consent. Seventeen right-handed students (women = 8) from a university in Korea aged between 20 and 29 years (M = 24.2, SD = 2.4) were paid to participate in the fMRI experiment. The G-power 3.1.9 software was used to verify the sample size. Our study included more participants than the number of samples required for power = 0.8 and α = 0.05 in the within-subject design.

#### 3.2.2. fMRI stimuli and procedure

At the beginning of the experiment, the participants read a brief introduction and viewed images related to that condition, as in Study 1 (Figure 2). We used an event-related design with two conditions: climate and control. The images for both conditions were the same as those used in Study1. Each condition contained ten images, and two trials were conducted for each condition. Each image was presented for 6 s and a fixation page with a cross sign at the center of the screen was projected for 1 s in between events.

The participants lay comfortably on a Siemens Prisma 3T scanner to record the structural and functional data. Their heads were fixed in place using foam blocks, ensuring later analysis would be accurate. All data on stimulation presentation and button responses were collected using Psychtoolbox 3.0 running on MATLAB. After the experiment was completed, the participants were given a small reward.

#### 3.2.3. Data acquisition and analyses

Structural images were acquired using a T1-weighted protocol: repetition time (TR) = 1000 ms; echo time (TE) = 2.28 ms; flip angle = 8°; field of view (FOV) = 240 × 240 mm^2^; voxel size = 3 mm isotropic. The acquisition parameters for task fMRI data were as follows: TR = 1000 ms; TE = 30 ms; flip angle = 90°; FOV = 252 × 252 mm; voxel size = 3 mm isotropic.

Data preprocessing and further neural data analysis were conducted using Functional MRI of the Brain software library (FSL 6.0; Jenkinson et al., 2012). First, functional data were preprocessed to remove sources of noise and artifacts and corrected for differences in slice acquisition time. Functional data were then normalized to a standard space (3 mm isotropic voxels) based on the MNI152_3mm template [Montreal Neurological Institute (MNI)]. Each GLM was convolved with a canonical hemodynamic response function (HRF) and used to generate a contrast image that compared the climate and control activation for each participant. These contrast images were collapsed across groups and entered into a second-level fixed-effects analysis that was thresholded at *p* < 0.05, with a Z threshold of 1.96 cluster activated voxels. This analysis resulted in a whole-brain statistical parametric map that identified the regions that displayed greater activity in response to climate stimuli than they did in response to control stimuli.

To investigate the differences between the climate and control conditions, we performed a region of interest (ROI) analysis. The parameter estimates for each participant were extracted based on anatomically defined ROIs from the Neurosynth database (Yarkoni et al., 2011). Parameter estimates were conducted to investigate the effects of stimulus activation on regional brain responses to climate (vs. control) images and paired-sample *t*-tests were used for statistical analysis.

#### 3.2.4. Results

**Figure 3:**
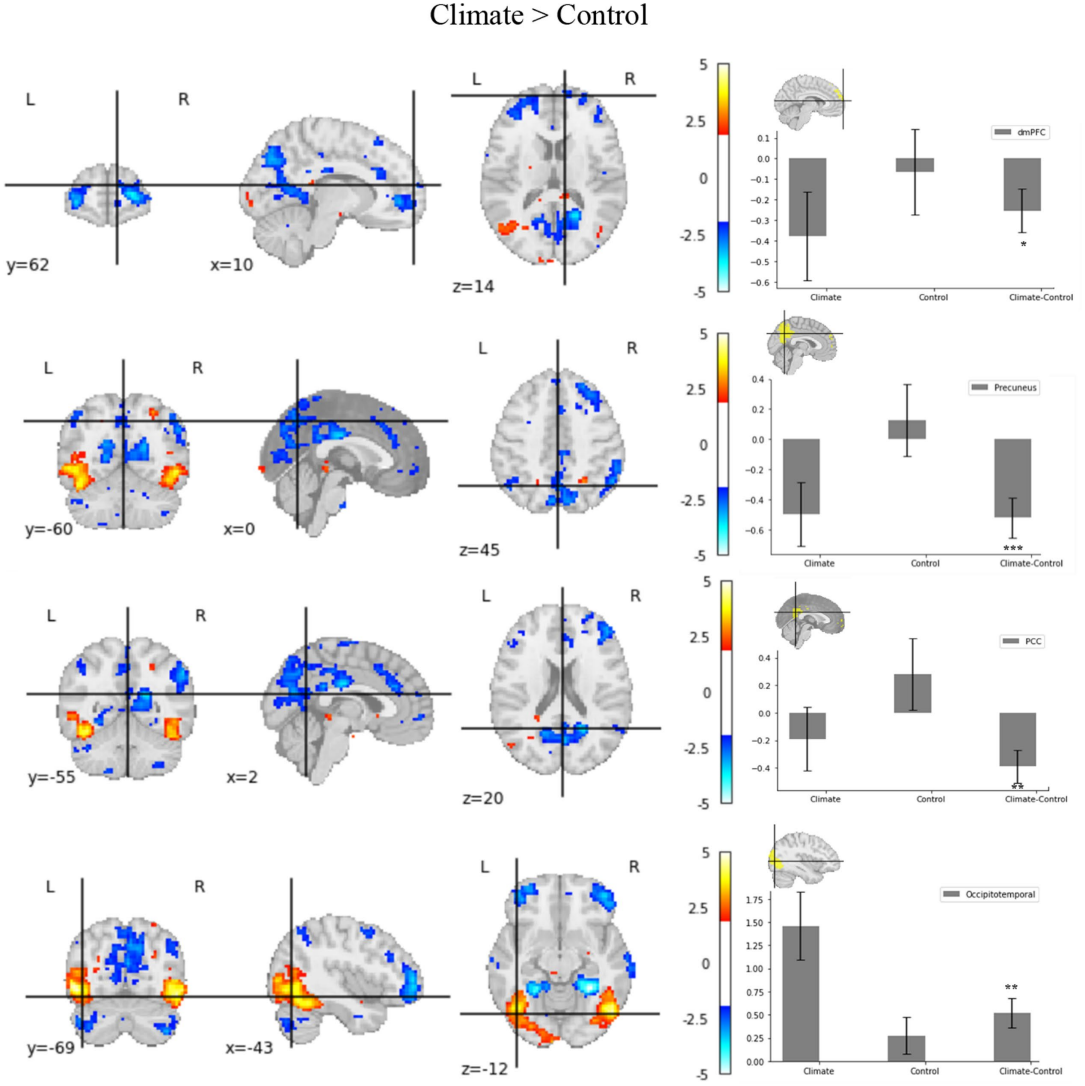
Whole brain map of differences between climate and control conditions. The left part of the figure shows the whole-brain map for contrasting climate conditions with a control condition (cluster z threshold of 1.96, *p* < 0.05, corrected). The dmPFC, precuneus, and PCC regions, associated with the self-referential process, showed decreased activation, whereas the occipitotemporal region, associated with the external attention process, showed increased activation. The right part of the figure shows the regions of interest (ROI) for self-awareness processing and the external attention areas under control and climatic conditions. Analysis of the difference scores derived from parameter estimates within the dmPFC (10, 62, 14), precuneus (0, −60, 45), and PCC (2, −55, 20) revealed decreased activity in response to climate images. Analysis of difference scores derived from parameter estimates for climate images relative to control images in occipitotemporal areas (−43, −69, −12) revealed increased activity, specifically for climate images. These coordinates were reported in the MNI stereotaxic space. **p < 0.01, ***p < 0.001. Bars indicate the standard error of the mean.

The fMRI data showed decreased activation in the dmPFC, precuneus, and PCC—all of which are engaged in self-awareness processes, when exposed to climate crisis messages (as opposed to control messages). Additionally, the occipitotemporal region, which is associated with external attention, was activated under climate change conditions (Table 1; Figure 3).

**Table 1:** Active brain regions for each condition in all participants.

Region	Cluster size (voxels)	*Z* score	MNI-coordinate		
			*x*	*y*	*z*
Climate > Control					
Middle Occipital Gyrus (L)	1,077	4.8	−48	−69	−6
Temporal Lobe, Fusiform Gyrus (L)		4.27	−39	−54	−18
Middle Occipital Gyrus (L)		3.53	−42	−84	3
Middle Temporal Gyrus (L)		3.38	−51	−66	9
Middle Occipital Gyrus (R)	683	5.01	51	−75	−6
Fusiform Gyrus (R)		4.67	51	−69	−12
Control > Climate					
Fusiform Gyrus (R)	1713	4.96	30	−45	−12
Posterior Cingulate (R)		4.07	21	−57	18
Limbic Lobe, Cingulate Gyrus (R)		3.56	3	−18	30
Occipital Lobe, Cuneus (L)		3.49	−6	−78	36
Parietal Lobe, Precuneus (R)		3.19	9	−75	39
Frontal Lobe, Superior Frontal Gyrus (R)	1,641	3.83	27	60	3
Middle Frontal Gyrus (R)		3.57	42	51	−15
Superior Frontal Gyrus (R)		3.42	36	15	51
Middle Frontal Gyrus (L)	591	3.81	−33	54	0
Superior Frontal Gyrus (L)		3.6	−27	60	−3
Medial Frontal Gyrus (L)		2.69	−21	48	12
Angular Gyrus (R)	333	3.52	57	−57	36
Inferior Parietal Lobule (R)		3.34	57	−54	42
Precuneus (R)		2.63	45	−72	36
Superior Temporal Gyrus (R)		2.61	57	−57	24

z threshold, 1.96; *p*, 0.05.

We conducted an ROI analysis to test our hypothesis that climate stimulus significantly decreases activation in the dmPFC, precuneus, and PCC and increases activation in the occipitotemporal region compared to control stimuli. We set these areas as the ROIs of the study and extracted the z-values. Participants’ brains showed decreased activation in the dmPFC (M_control_ = −0.06 [SD = 0.85], M_climate_ = −0.38 [SD = 0.89], *t* (16) = −2.4, *p* < 0.05), precuneus (M_control_ = 0.13 [SD = 0.98], M_climate_ = −0.50 [SD = 0.86], *t* (16) = 4.01, *p* < 0.001), and PCC (M_control_ = 0.28 [SD = 1.05], M_climate_ = −0.19 [SD = 0.96], *t* (16) = 3.47, *p* < 0.01) areas engaged in self-awareness when they were exposed to climate crisis messages compared to control messages (Figure 3). Therefore, H2 is supported. Participants’ brains also showed increased activation in the occipitotemporal areas engaged in the external attention process when exposed to climate crisis messages compared to control messages (M_control_ = 0.93 [SD = 1.25], M_climate_ = 1.49 [SD =1.53], *t* (16) = 3.21, *p* < 0.01; Figure 3). Therefore, H3 is supported.

**Figure 4:**
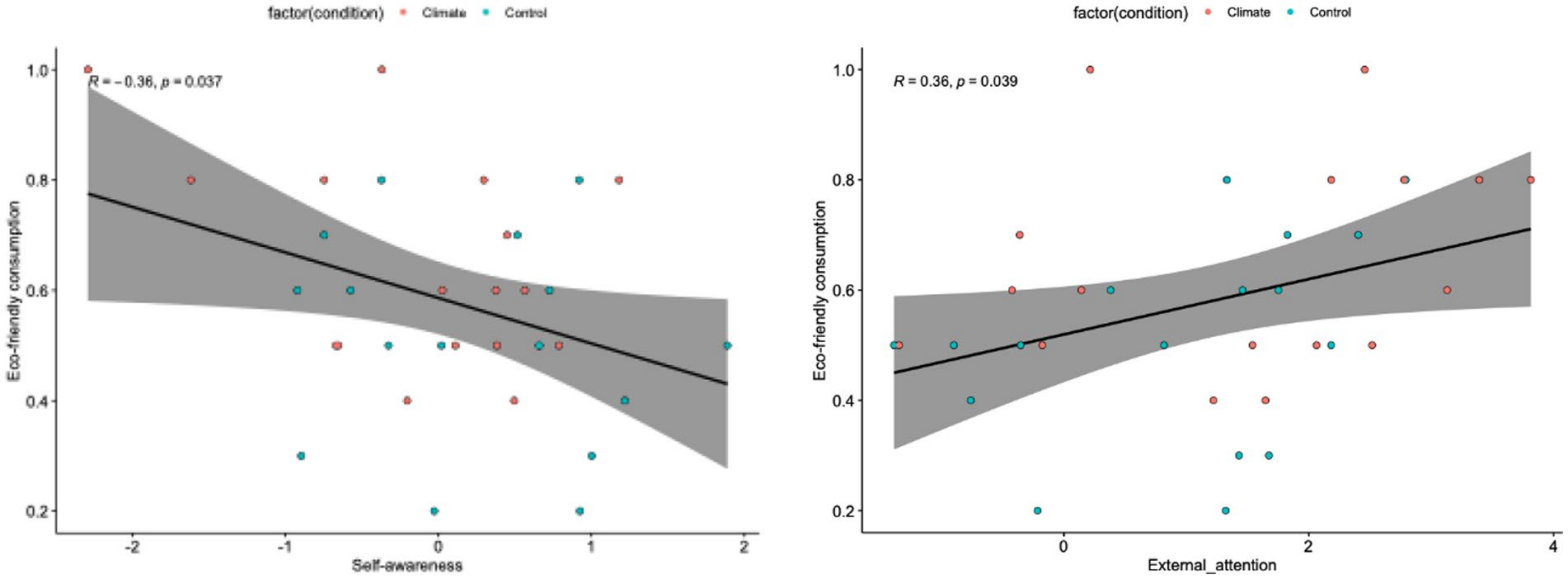
Correlation between self-referential/external attention process and eco-friendly consumption. Correlations between ROIs and eco-friendly consumption. The left part of the figure shows the relationship between eco-friendly consumption and parameter estimates of self-referential processing, including the dmPFC, precuneus, and PCC. The right side of the figure shows the relationship between eco-friendly consumption and the parameter estimates of external attention processing, including the occipitotemporal region.

We compared keypad responses collected during the fMRI experiment using a paired *t*-test (M_control_ = 0.51 [SD = 0.19], M_climate_ = 0.65 [SD = 0.19], *t* (16) = 2.75, *p* < 0.05). Behavioral data showed that participants chose more eco-friendly products under climate conditions. We also investigated the relationship between eco-friendly product selection and the estimated parameter data and found that the correlation between the self-awareness process and the eco-friendly selection ratio based on the two conditions was significant (*r* = −0.36, *p* < 0.05; Figure 4), such that the activation of the self-awareness process decreased, and eco-friendly consumption increased. Similarly, the estimated parameter data of the occipitotemporal region, engaged in the external attention process, were significant (*r* = 0.36, *p* < 0.05; Figure 4) such that the larger the activation of the external attention process, the more eco-friendly consumption will be.

#### 3.2.5. Discussion

In the fMRI study, we found that participants had decreased activation of the dmPFC, precuneus, and PCC areas and increased activation of the occipitotemporal areas in climate than in control conditions. The dmPFC, precuneus, and PCC are involved in self-referential processing (Zhang and Chiang-shan, 2012). Decreased activation of self-awareness areas and increased activation of external attention areas suggests that an individual’s reference has shifted from the self to the environment, supporting the “awe” theory. Additionally, the relationship between the estimated parameter data of the self-awareness and external attention areas and eco-friendly consumption was significant. This suggests that the neural indicators reported in this study can reflect future behaviors. Therefore, it is important to experience awe to increase eco-friendly consumption. Our results reveal how the brain functions during human exposure to climate change messages, and how neural activity in the brain relates to subsequent behavioral choices.

## 4. General discussion

As environmental issues receive widespread attention and concern in the public arena, consumers agree with eco-friendly consumption attitudes; however, there is a gap between this attitude and behaviour (Carrington et al., 2010; Zhang et al., 2022). This indicates the necessity of promoting sustainable consumption by increasing consumer awareness of environmental issues through effective communication. Previously, activation of the dopamine-based reward circuit, that is, the pursuit of personal satisfaction by consumers, was considered an achievement in marketing activities (Zhang and Lee, 2022). However, in the future, marketing should pay more attention to the activation of cool and calm psychological circuits, such as ecological consciousness, rather than satisfying individual needs. This study investigated the psychological mechanism of awe for eco-friendly consumption by examining behavioral and blood-oxygen-level-dependent (BOLD) responses. Therefore, this study is one of the first attempts to identify awe based on behavioral and biological methodologies and promote sustainable behaviour.

Exposure to climate change cues is associated with many psychological benefits (Lange and Truyens, 2022), so our experiment investigated the psychological processes that occur when people are exposed to climate change messages. Exposure to climate change cues caused individuals to pay attention to climate messages and induced awe experience compared with control cues (Studies 1 and 2). Additionally, we tested the recorded brain activity and subsequent behaviour after exposure to climate change messages. Based on the ROI analysis, exposure to climate crisis messages decreased the activation of the dmPFC, precuneus, and PCC—all areas that engage in self-referential processing (Study 2). The analysis of the ROI also showed that exposure to climate crisis messages activated the occipitotemporal areas that belonged to external attention (Study 2). Thus, the behavioral and fMRI experiments support our theoretical explanation that the psychological mechanism underlying the effect of climate change on eco-friendly consumption is the experience of awe.

The awe ratings indicate that our experimental design can induce awe. The participants in the climate crisis condition experienced a greater sense of awe than those in the control condition. Previous studies have reported similar effects of task type on the subjectively experienced sense of awe (Greicius and Menon, 2004; McConnell and Jacobs, 2020). This experiment provides a reliable way to experience awe. Our findings support the suggestion that environmental messages are characterized by reduced self-awareness, which is reflected in reduced DMN activity.

Exposure to messages about climate change can promote eco-friendly consumption. This finding is consistent with previous studies showing that exposure to nature images increases an individual’s environmental awareness and engagement in eco-friendly behaviour (Klein and Hilbig, 2018; Coughlan et al., 2022). From an individual’s perspective, environmentally friendly behaviour benefits the community and requires individual sacrifice (Piff et al., 2015). Therefore, climate change messages are also a useful approach for promoting the need to protect the environment and increase cooperative actions that benefit communities. This sustainability communication can help awaken the public and promote sustainable consumption of all species—including humans, animals, and plants—and minerals.

The results of this study have several theoretical implications. First, when exposed to climate change cues, people shift from self-centric to eco-centric processes. The DMN, which includes the dmPFC, precuneus, and PCC, is involved in self-centric processing and is an integrated system for self-related cognitive activities including autobiography, self-monitoring, and social cognitive functions (Spreng et al., 2009). Decreased activation of the self-awareness process reduces the self-centric perspective and pays attention to external events (Scheibner et al., 2017). The activation of the occipitotemporal region upon exposure to climate change suggests that climate change is related to environmental attention processing. The occipitotemporal region is involved in external attention, indicating that climate change is oriented toward highly focused and environmentally oriented information processing. This region encodes objects so that individuals can recognize them and cooperate with the environment (Epstein, 2005). Our results are significant in reducing self-centric processing and increasing eco-centric processing related to climate change.

Second, climate change messages induce consumers to switch from conventional to eco-friendly products. This eco-centric perspective shows that consumers prefer eco-friendly consumption, even at a personal cost, suggesting that exposure to climate change messages shifts humans from being self- to eco-centered by recognizing the value of the environment. Existing marketing research emphasizes self-enhancement and seeks to address desires, such as power and achievement. However, self-enhancement can also lead to negative consequences related to the pursuit of social status (Cannon and Rucker, 2022). Focus on ourselves makes us so delicate, sensitive, and fragile that every experience is not positive. To solve the problem of sustainability, we must move away from ourselves and serve the environment to improve the world (Frantz et al., 2005). Our results suggest that marketing communications related to nature encourage people to realize the value of the environment for themselves and shift from being human- to ecological-centered.

This study has several practical implications for marketers and policymakers. First, exposure to climate change messages induces individuals to switch to eco-friendly products because it suggests that they think less about their self-interest. To promote eco-friendly consumption, policymakers should use mechanisms such as eco-centric approaches for communication. Second, the results of this study allow marketers to understand consumer behaviour using a brain map so that they can understand how to satisfy consumers’ diverse needs in a way that leads to sustainable consumption. Using this brain map, marketers can develop specialized and tailored programs to encourage sustainable consumption among customers who may otherwise resist it.

Our study had several limitations. To increase sustainable behaviour, we use climate images as stimuli to induce awe in consumers. However, factors that increase sustainable behaviour include both other-oriented emotions, such as awe, and self-oriented emotions, such as pride or self-restoration. Sustainable consumption may lead to differences in consumers’ purchase intentions depending on the context; therefore, it is necessary to induce emotions that are appropriate to the situation by expanding various sets of emotions. Additionally, human neuroscience experiments often have limited sample sizes, reducing the generalisability of the results. Future studies should use larger sample sizes to increase generalisability. Finally, when examining the brain pathways related to exposure to climate change messages, we focused on self-awareness and external attention processing. Future studies should investigate larger regions of the brain, such as the limbic areas. These areas may be associated with social collaboration efforts toward the goals of environmental, social, and corporate governance (ESG; Alsharif et al., 2021). We look forward to a more in-depth investigation of the transition from themselves to an eco-centric perspective in future studies.

## 5. Conclusion

We have been living with climate change for a long time and are aware of its dangers (Berrang-Ford et al., 2011). In response to the climate crisis, macro-level policies, such as transitioning to renewable energy are being implemented. Simultaneously, individual consumers are being called upon to participate in more eco-friendly consumption movements. Consumers who are aware of the climate crisis may find that eco-friendly consumption, as a form of consumer activism, often entails personal sacrifices. For instance, electricity generated from renewable sources may be more expensive than that produced from fossil fuels, requiring consumers to accept higher costs for fewer short-term benefits. In the tradition of economic knowledge stemming from Smith (1937[1776]), individuals are believed to pursue their own interests with selfish motivation for economic activities. However, the market’s invisible hand optimally coordinates these actions. In the context of the climate crisis, there is a growing awareness that individuals must set aside their selfish gains and actively participate in collective, cooperative, and mutually adaptive efforts to save the planet, emphasizing the importance of a unified direction and moving toward a sustainable relationship with Earth and Mother Nature. In this study, we proposed a need to shift toward a more holistic view of oneself as a coexisting partner of Earth and Mother Nature, instead of living at their expense. This study investigated how eco-friendly consumption can be induced by highlighting the neural basis of exposure to climate change messages based on the human-environment theory. Self-enhancement helps to meet individual needs, but it can become an obstacle in the current area of sustainable consumption.

We used behavioral and fMRI experiments to uncover the consumer’s hidden process: awe. This study showed that an individual’s awe experience was accompanied by decreased activation of self-awareness processing and increased activation of external attention processing. If the participant selected an unwanted eco-friendly product according to social desirability, the dorsolateral prefrontal cortex (DLPFC) related to social preference or the anterior cingulate cortex (ACC) related to cognitive conflict/dissonance will be activated (Sawe and Chawla, 2021). However, in our study, we know that environmental cues are powerful visual representations that induce an awe experience accompanied by reduced self-referential processing and facilitate eco-friendly behaviour. We suggest that awe promotes eco-friendly consumption, indicating the need for humans to escape the self and experience awe.

## Data availability statement

The original contributions presented in the study are included in the article/supplementary material, further inquiries can be directed to the corresponding author.

## Ethics statement

The studies involving humans were approved by the Institutional Review Board (IRB) of Sungkyunkwan University (2021-12-018). The studies were conducted in accordance with the local legislation and institutional requirements. The participants provided their written informed consent to participate in this study.

## Author contributions

MY methodology, writing, and editing. E-JL project administration, review and editing. All authors contributed to the article and approved the submitted version.

## Funding

This research is funded by Korea National Research Foundation (NRF) (2021R1A2B5B01001391) awarded to E-JL.

## Conflict of interest

The authors declare that the research was conducted in the absence of any commercial or financial relationships that could be construed as a potential conflict of interest.

## Publisher’s note

All claims expressed in this article are solely those of the authors and do not necessarily represent those of their affiliated organizations, or those of the publisher, the editors and the reviewers. Any product that may be evaluated in this article, or claim that may be made by its manufacturer, is not guaranteed or endorsed by the publisher.

## Figures and Tables

**Table:** 

Vastness vis-a-vis the self scale Piff et al. (2015)	I feel the presence of something greater than myself. I feel part of some greater entity. I feel like I am in the presence of something grand. I feel like I am a part of a greater whole. I feel the existence of things more powerful than myself.
Self-diminishment scale Piff et al. (2015)	I feel small or insignificant. I feel like my own day-to-day concerns are relatively trivial. In the grand scheme of things, my own issues and concerns do not matter as much. I feel insignificant in the grand scheme of things. I feel small relative to something more powerful than myself.

## Appendix

Measurements used in Study 1.
